# Artificial intelligence and clinical reasoning - a qualitative study with general practice trainees in Germany

**DOI:** 10.1186/s12909-026-10736-3

**Published:** 2026-11-14

**Authors:** Andreas Christian Dreher, Hendrik Sauer, Attila Altiner, Simon Schwill

**Affiliations:** Department of General Practice and Health Services Research, University Hospital Heidelberg, Heidelberg, Germany

**Keywords:** Artificial intelligence, General practice, Clinical reasoning, Postgraduate medical education, Large language models

## Abstract

**Background:**

General practitioners face high complexity in clinical decision-making. Large language models (LLMs) may support clinical reasoning, but their impact on decision-making processes and postgraduate medical training remains unclear. This qualitative study aims to explore General Practice (GP) trainee’s use of LLMs in clinical reasoning and their educational needs.

**Materials and methods:**

GP trainees were recruited and interviewed with semi-structured interview-guide. Interviews were transcribed verbatim and analysed qualitatively.

**Results:**

Seventeen GP trainees participated (mean interview duration: 38 min; range: 21–63 min). AI was perceived as a tool to augment and accelerate clinical decision-making, while potential risks of deskilling were identified. Participants reported a lack of structured AI education and expressed a need for its integration into postgraduate training.

**Discussion:**

Participants perceived AI primarily as a supportive tool that can complement clinical reasoning and improve access to medical knowledge. At the same time, they identified potential risks, including overreliance on AI-generated information and possible effects on the development of clinical expertise. These findings suggest that postgraduate medical training should address AI-related competencies, including critical appraisal, ethical responsibility and appropriate integration of AI into clinical decision-making.

**Conclusion:**

AI is already part of clinical practice among GP trainees, offering opportunities to support learning and clinical reasoning while raising concerns about overreliance and critical appraisal. Postgraduate medical training in GP should therefore incorporate structured AI competencies to ensure responsible, reflective and patient-centered use of AI.

**Supplementary Information:**

The online version contains supplementary material available at https://doi.org/10.1186/s12909-026-10736-3.

## Introduction

General practitioners (GPs) manage complex clinical demands driven by population ageing, multimorbidity, workforce shortages, administrative burden and the rapid expansion of medical knowledge [1]. Clinical reasoning (CR) [2] and critical appraisal are therefore essential competencies for delivering high-quality care under conditions of uncertainty and incomplete information [3]. CR in general practice (GP) is characterised by low disease prevalence at the point of first presentation, limited diagnostic options, undifferentiated presentation of symptoms and the integration of patients’ biopsychosocial contexts [4]. This is why clinical reasoning in GP particularly depends on the integration of heterogeneous information, contextual knowledge and probabilistic decision-making. In this context, large language models (LLMs) have emerged as potential tools to support physicians in managing information complexity, documentation demands and clinical decision-making in primary care [5–9]. In primary care, where physicians frequently evaluate early and nonspecific presentations, LLMs may support the recognition of uncommon conditions [10]. However, they may also contribute to unnecessary diagnostic cascades and overtreatment. Despite their potential benefits, the implementation of LLMs raises ethical and practical concerns, including inaccurate or hallucinated outputs, data governance, privacy, liability and environmental sustainability [6]. Furthermore, research with AI applications for cardiovascular risk prediction, skin cancer detection and chronic disease management [11, 12] shows promising results. For now, many AI systems remain unsuitable for routine clinical use because of insufficient validation, limited transparency and biased training data [13, 14].

GPs already need to manage complexity and navigate uncertainty [15] to deliver high-value care. LLMs introduce a new form of cognitive support that may influence how trainees acquire, apply and reflect on clinical reasoning skills. Little is known about how GP trainees use LLMs for clinical reasoning and which educational challenges and learning needs emerge from their use [16, 17].

This qualitative study aims to explore how GP trainees integrate LLMs into clinical reasoning, how they reflect on their use and its ethical implications, and which educational needs they identify for postgraduate medical training.

## Materials and methods

### Study design and setting

We conducted a qualitative study interviewing GP trainees with the help of semi-structured interviews. A qualitative approach was chosen to enable an in-depth exploration of experiences and perceived challenges with the use of AI in GP. Recruitment, interviews and subsequent data analysis were carried out over a 16-week period between March 2026 and June 2026. Reporting of the study followed the Consolidated Criteria for Reporting Qualitative Research (COREQ) guidelines (see Appendix 1) [18].

### Participants and recruitment

All participants were physicians specialising in GP (5 years of training) and working in a outpatient clinical GP rotation at the time of the study. Trainees working in an inpatient rotation were excluded. In Germany GP training is five years full-time training structured by the medical association´s model speciality training regulations with a 12-month in-patient rotation in internal medicine, a 24-month rotation in GP, a 6-month rotation in a third patient-centred medical discipline like pediatrics, psychiatry or dermatology as well as 18 months of rotation in further subdisciplines or internal medicine. The German postgraduate medical training could be described as an individual apprenticeship without compulsory institutional training.

All participants were enrolled in the Competence Centre for Postgraduate Medical Education Baden-Württemberg (KWBW). Baden-Württemberg is a federal state in Germany with approximately 11 million inhabitants. The KWBW supports structured postgraduate training in GP with seminar programmes alongside on-ward training under supervision, a mentoring-programme and train the trainer-courses [19]. The German healthcare system is based on a social health insurance model with statutory health insurance covering the majority of the population alongside private health insurance. General practice plays a central role in primary care, providing first-contact medical care, managing common health conditions and coordinating patients’ care across different healthcare settings, although access to specialist care is not generally restricted by a mandatory gatekeeping system. We used purposive sampling, specifically criterion-based sampling, to recruit participants meeting the study’s eligibility criteria while seeking variation in AI experience, stage of postgraduate training, age and gender. Recruitment was facilitated through KWBW seminars, from which interested trainees were invited to participate. Some participants were known to the researchers through professional networks. However, no hierarchical or other dependent relationship existed between researchers and participants. This relationship did not influence participation, data collection or data interpretation. A total of 48 trainees were contacted, of whom 23 responded and 17 ultimately participated in an interview.

### Development of the interview guide

Following a literature review (NIH/PubMed^®^ search terms: “artificial intelligence”, “large language models”, “clinical reasoning”, “clinical thinking”, “decision making”, “general practice” and “postgraduate medical education”), an interview guide was developed by a team of three researchers (ACD, HS and SS). HS was GP trainee and academic researcher with substantial experience in qualitative research, ACD a GP trainee and board certified specialist for psychosomatic medicine with years of experience in qualitative research and SS, a consultant for general practice, Master of Medical Education with many years of experience in qualitative research.

Kern’s six-step approach to curriculum development served as a theoretic framework for the development of the interview guide, focussing the assessment of educational needs among GP trainees [20]. The development of the interview guide focused on understanding how GP trainees integrate LLMs into clinical reasoning and identifying their educational needs.

A semi-structured interview format was chosen to ensure systematic coverage of the key thematic domains while simultaneously allowing free associations and follow-up questions.

The comprehensibility and clarity of the interview guide were assessed using the think-aloud technique [21], in which participants verbalise their cognitive processes, strategies and decision-making while responding to questions. Subsequently, the interview guide was piloted with two interviewees, which were not included in the study sample and the interview guide was revised slightly (see Appendix 2). The final interview guide included questions on sociodemographic characteristics, experiences with large language models (LLMs) in clinical reasoning, perceived influences of LLM use on managing clinical uncertainty, ethical considerations and educational needs for postgraduate medical training.

### Data collection

Interviews were conducted via Zoom^®^ (San Jose, California, USA; version 6.3.0) by HS. At the beginning of each interview, participants were informed about the background of the study and the role of the researcher. No third parties (non-participants) were present either at the researcher’s workplace or in the private environments of the participants during the interviews. Audio recordings were made for all interviews. Field notes were taken to document relevant observations made during data collection. Interview transcripts were not returned to participants for commenting or correction. The dataset was considered sufficiently rich to answer the research questions. During the final interviews, no substantial new themes emerged.

### Qualitative methodology and data analysis

We chose a qualitative descriptive approach with the aim to explore data-near and comprehensivly GP trainees´ experience and perceptions regarding AI usage and its perceived influence in clinical thinking.

The audio recordings were transcribed verbatim using MacWhisper version 13.2 (Argmax), running the NVIDIA Parakeet v3 speech recognition model locally on-device. The transcripts were subsequently checked by the interviewer for completeness and accuracy. Data was managed using Microsoft Excel 2024 (Microsoft Corporation, Redmond, WA; USA) and analysed manually. Transcripts were reviewed and coded using an qualitative content analysis approach based on Kuckartz [22]. We selected Kuckartz´s qualitative content analysis as it provides a systematic data-near procedure for developing und differentiating thematic categories across heterogeneous interviews. Kuckarzt requires thematic coding and uses a inductive-deductive approach. Main categories were derived deductively from the research questions and were included in the interview guide. During the analysis, additional subcategories and codes were added inductively based on the interview material. If new subcaterogies or codes were found later on in the progress, all previous interviews were analysed again. HS and ACD independently analysed the material. Categories and representative quotations were subsequently compared and revised where necessary. In cases of disagreement or uncertainty, a third researcher (SS) reviewed the material to reach consensus. Reflexivity was maintained throughout analysis by considering how researchers’ clinical and personal backgrounds, prior experiences with AI and professional perspectives could influence interpretation [23].

### Ethical approval

The study was part of a larger cohort-study approved by the Ethics Committee of Heidelberg University Hospital (Nr. S-570/2015). All participants provided written informed consent prior to participation.

## Results

A total of 17 interviews were conducted between March 2026 and June 2026. The mean interview duration was 38 min (range: 21–63 min). No relevant technical problems occurred during data collection and none of the interviews had to be interrupted nor repeated.

**Fig. 1:**
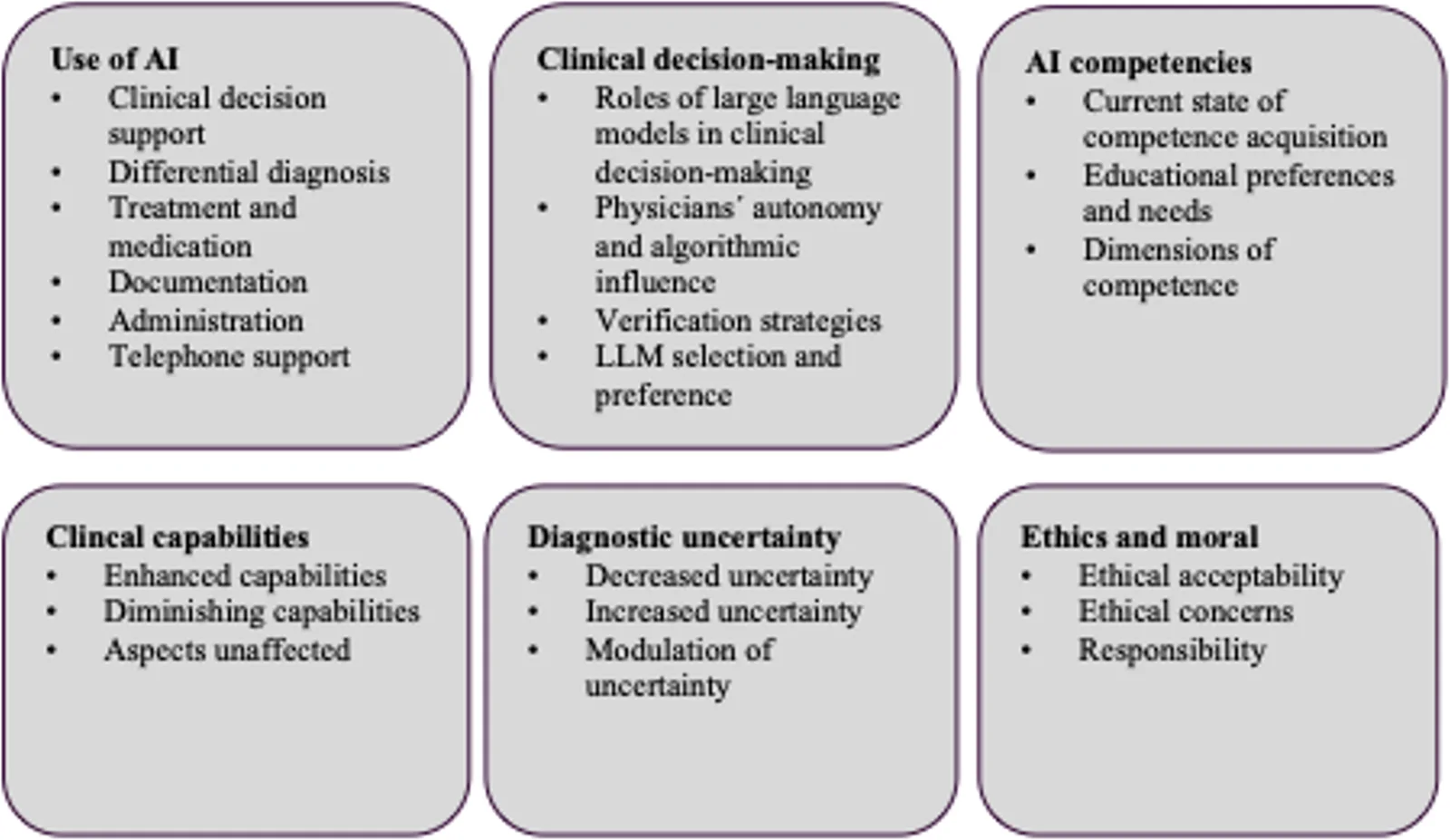
Overview of the main categories and subcategories and use of AI

The coding framework was reviewed after the first two interviews and no major modifications were required. No new themes or codes emerged in the final interviews, indicating that meaning saturation had been achieved and no additional participants were recruited. The analysis yielded five main categories, 17 subcategories and 58 codes which are shown in Tables 2, 3, 4, 5 and 6. Figure 1 shows an overview of the identified themes. The sociodemographic characteristics are shown in Table 1. No participant reported never using AI.

**Table 1:** Sociodemographic data of the participating general practice trainees

*n* = 17		Total
Gender	Female	8 (47.1 %)
	Male	9 (52.9 %)
Age in years	Median	34 (IQR = 29; 41)
Postgraduate training year	Mean	3.8 (SD = 0.75)
	Median	4 (IQR = 2; 5)
Frequency of AI use	Daily	8 (47.1 %)
	Several times a week	7 (41.2 %)
	Once a week	1 (5.9 %)
	Less often than once a week	1 (5.9 %)
	Never	0 (0%)
Interview duration	Minimum duration	20 min 41 s
	Maximum duration	1 h 03 min 05 s
	Total duration	10 h 51 min 57 s
	Mean	38 min 21 s

### Use of AI

AI was primarily used as an individual clinical decision-support tool rather than as an integrated component of practice workflows. The most frequently reported application was generating differential diagnoses or exploring diagnostic possibilities in clinically ambiguous cases. Other common clinical use cases included medication-related queries, such as dosing, treatment alternatives or drug comparisons, as well as use as a rapid reference or memory aid.

Beyond direct clinical decision support, participants used LLMs for drafting and revising clinical or administrative documents, including medical certificates, reports and applications. Personal, non-clinical use was also common, whereas image analysis of anonymized skin findings, AI-assisted translation during consultations and learning support were reported only occasionally. Three participants described their overall AI use as infrequent and cautious.

ChatGPT and the AI features integrated into Amboss ^®^ were the most used tools, followed by AI-generated Google search summaries. Other LLMs, including Gemini, Perplexity, OpenEvidence and Claude, were mentioned only occasionally.

Practice-level implementation remained limited. Telephone assistance and practice management systems represented the most frequently reported AI applications, whereas AI-supported documentation or voice assistants and billing or coding tools were reported less often. Seven practices reported no integrated AI solutions. AI use occurred predominantly through personal devices and freely available applications. Most participants did not hold a paid subscription.

Exploratory comparisons across gender suggested some tendencies in how interviewees described their use of AI, although no consistent pattern emerged overall. Female participants more often mentioned checking AI-provided sources and critically evaluating its responses, while male participants more frequently raised concerns about prompt quality and plausible but inaccurate outputs.

### Impact of large language models on clinical decision-making and evaluation of outputs

This category describes how AI becomes embedded in the clinical decision-making process and how physicians appraise its outputs. We identified four subcategories: the roles that large language models assume in clinical reasoning, the extent to which physician autonomy is preserved or eroded by algorithmic influence, the strategies used to verify AI-generated content and the preferences that guide the choice between general-purpose and specialized models.

#### Roles of large language models in clinical decision-making

A recurring pattern across interviews was the use of LLMs as cognitive support tools for generating additional perspectives and diagnostic hypotheses in clinically ambiguous situations. Participants described using LLMs to expand their own reasoning process rather than to replace clinical judgment.

#1: “Often […] to get differential diagnoses. […] To see, what have I already thought of, what haven’t I thought of yet. […] So I let myself be inspired, but I don’t let myself be persuaded.”

Closely related was the use of LLMs as a cognitive cross-check or second opinion. Participants typically described a sequential approach in which they first developed their own diagnostic considerations and subsequently used the LLM to verify or challenge their assumptions. Several participants reported using LLMs as an accessible source of reassurance or consultation, particularly when immediate discussion with colleagues or senior physicians was not possible in everyday practice. Others emphasised that LLMs did not fundamentally alter their clinical decisions but rather accelerate existing processes by facilitating information retrieval and summarisation. In addition, participants described LLMs as helpful in identifying additional aspects of a situation or suggesting further diagnostic considerations, such as additional laboratory tests.

### Physicians’ autonomy and algorithmic influence

Most participants consistently stated that responsibility for clinical decisions remained with the physician. LLMs were described as supportive tools and were not regarded as autonomous decision-making systems.

#16: “If AI gives me an answer, I would never simply adopt it or rely on it. […] So I just need to have seen it, but I make the decision.”

Participants identified several factors that could influence the degree of reliance on LLM-outputs. The broad scope of GP and limited access to immediate collegial exchange were described as factors increasing both the perceived usefulness of LLMs and the risk of accepting suggestions without sufficient cross-checking. Some participants perceived younger or less experienced physicians as potentially more susceptible to overreliance on AI-generated recommendations. In addition, some participants highlighted the persuasive nature of fluent and plausible-sounding responses as a potential source of bias. Three participants explicitly acknowledged having had a case where they were at risk at getting strongly influenced by outputs. Experiences with inaccurate or unreliable outputs were common. Most participants reported personal experiences with hallucinations, including incorrect information, contradictions or fabricated references. Only few participants reported encountering rare or no hallucinations during their routine clinical use.

### Verification strategies

Participants described several strategies to evaluate and verify LLM-generated outputs. The most frequently reported approach was comparison with established clinical resources and guidelines. Participants pointed out that their own medical expertise as an essential mechanism for assessing plausibility and identifying errors.

#15: “In order for me to notice that it’s wrong, I have to have a certain amount of knowledge.”

Other verification approaches included checking the sources provided by LLMs, iteratively refining prompts and questioning the LLM about its responses and disregarding outputs when contradictions could not be resolved. Participants frequently described verification as dependent on their own prior knowledge and clinical expertise.

### Large language model selection and preference

Participants reported differing preferences regarding LLM selection. Some participants perceived general-purpose models such as ChatGPT as faster and more helpful than dedicated medical AI tools, whereas others expressed greater trust in curated systems with defined source bases.

#8: “Well, I think Amboss itself is great, but I don’t think Amboss’s AI is nearly as mature as ChatGPT. […] I find it easier and faster with ChatGPT than with the Amboss AI with my medical background.”

Corresponding subcategories are presented in Table 2.

**Table 2:** Perceived role of large language models in clinical decision-making and evaluation of AI-generated outputs: subcategories, codes and anchor quotes from general practice trainees

**Code**	Description	Anchor Quote
Roles of large language models in clinical decision-making		
AI as a source of ideas	AI is used to provide additional perspectives, ideas, and thought-provoking impulses (e.g. differential diagnoses) for decision-making. (n = 14: #1, #2, #3, #4, #5, #6, #8, #9, #10, #12, #13, #14, #15, #17)	*#1: "Often [...] to get differential diagnoses. [...] To see, what have I already thought of, what haven't I thought of yet. [...] So I let myself be inspired, but I don't let myself be persuaded."*
AI as a control authority	AI serves to verify assessments that have already been formed. Reassurance from the AI instead of from the postgraduate trainer. (n = 9: #1, #5, #6, #9, #10, #12, #14, #16, #17)	*#12: “I had already given it some thought beforehand and really just want it confirmed. [...] I mean, because you’re on your own and don’t have a senior physician at hand, you’re actually more inclined to ask AI. In the hospital you just call the senior physician.”*
AI to accelerate decision-making	AI is used to make information summarizing and research processes more efficient. AI speeds up decision-making. (n = 4: #1, #2, #5, #13)	*#2: “I don’t think it greatly influences my decision-making, but it does speed it up.”*
Physicians´ autonomy and algorithmic influence		
Decision-making authority remains with the physician	AI is understood as a support tool, not a decision-making tool; the clinical decision explicitly remains with the physician. (n = 14: #1, #2, #3, #5, #6, #7, #8, #10, #12, #13, #14, #15, #16, #17)	*#16: “If AI gives me an answer, I would never simply adopt it or rely on it. [...] So I just need to have seen it, but I make the decision.”*
Increased influenceability of general practicioners	The broad scope of the specialty and the lack of immediate collegial consultation increase the usefulness of AI, but also the temptation to adopt answers uncritically. (n = 7: #2, #8, #9, #11, #12, #13, #14)	*#2: “Yes, because general practitioners are still often […] lone wolves. […] I think the temptation is considerably higher to just accept that answer when you don’t know it yourself than when you can still discuss it critically with a colleague.”*
Increased influenceability of inexperienced physicians	Younger/inexperienced doctors are more strongly influenced by AI. (n = 4: #2, #6, #14, #16)	*#14: "I think the young generation is very influenced (by AI). And I think my generation is already using it a lot."*
Danger of uncritical adoption	Perception that AI can lead to greater adoption of their suggestions through plausible-sounding answers; three participants admit to their own strong influence. (n = 7: #2, #5, #8, #9, #11, #14, #16)	*#11: "If a question is asked to the AI, the answers are already very elaborate, then you tend to rely on it and then question it less critically."*
Experience with hallucinations	Own experiences with hallucinations/false or contradictory information; in particular, invented sources/DOIs as a typical form of error. (n = 12: #2, #3, #4, #6, #9, #10, #12, #13, #14, #15, #16, #17)	*#4: "Personally, I have also noticed again and again that the AI simply hallucinates and then sometimes [...] that there is actually no evidence for this now, but that it thought it could fit together quite well."*
No experience with hallucinations	Little/no hallucinations noticed in one's own medical routine. (n = 5: #2, #5, #7, #8, #11)	*#8: "actually much less than I thought [...] I'm impressed by how accurate (AI) is."*
Verification strategies		
Verification based on one’s own expertise	Own medical knowledge serves as a filter for evaluating the AI proposals; Recognizing mistakes requires prior knowledge (only ask what you can judge for yourself). (n = 9: #2, #3, #5, #6, #7, #8, #12, #15, #16)	*#15: "In order for me to notice that it's wrong, I have to have a certain amount of knowledge."*
Verification using standard sources	Dealing with contradictions: Comparison with guidelines/standard sources (Amboss, Deximed, Herold, Fachinfo, DEGAM). (n = 12: #1, #3, #5, #6, #7, #8, #10, #13, #14, #15, #16, #17)	*#16: „[...] I still have to validate it with a source I know and trust."*
Verification using the sources provided by AI	Checking the sources indicated. (n = 6: #2, #7, #11, #14, #15, #17)	*#17: "I'll read it through and then actually check it again and look at the sources.*
Verification by asking AI	Ask, prompt specificly, "discuss" with the AI. (n = 6: #5, #6, #8, #9, #14, #15)	*#6: "And sometimes I discuss when I have time [...] sometimes with the AI and say: okay, yes, but I know it this way and that. What do you think of that?"*
Discard the AI results in case of contradiction	If there is a persistent contradiction, the result of the AI will be discarded. (n = 4: #9, #11, #15, #16)	*#16: "I definitely trust my previous knowledge. Then I just say to myself, that's nonsense, what the AI says, honestly."*
Large language model selection and preference		
Open models more helpful than specific models	Open models (ChatGPT) are perceived as faster/more helpful than specific AI (Amboss). (n = 3: #2, #8, #10)	*#8: "Well, I think *Amboss* itself is great, but I don't think *Amboss's* AI is nearly as mature as ChatGPT. […] I find it easier and faster with ChatGPT than with the *Amboss* AI with my medical background."*
More confidence in specific models	Curated AI (Amboss) is more trusted than open models. (n = 3: #3, #6, #16)	*#16: "if I use the *Amboss AI now, for example, [...] that's its own platform, if I ask the AI, it only uses its own Amboss sources."

### Competencies and learning concepts for an appropriate use of large language models

This category addresses the competencies physicians consider necessary for the proper use of AI and how these can be acquired. The subcategories distinguish the current state of competence acquisition, the educational preferences and needs derived from it and the distinct dimensions of competence that together constitute the skill set required for safe and effective AI use.

#### Current state of AI-related competence acquisition

A central finding was a mismatch between current AI-related skills and participants’ perceived need for structured preparation. Participants reported that their knowledge and skills had largely been acquired informally through experimentation, learning by doing, testing different models and peer exchange.

#6: “A lot of learning by doing, I would say […] to deal with AI, to try out different ones, to use different large language models.”

Formal educational opportunities were rarely encountered. Half of the participants expressed a desire for structured AI training during medical school and postgraduate medical education, with some advocating mandatory integration into curricula.

#### Educational preferences and needs

Participants consistently preferred practice-oriented, case-based and interactive educational formats over traditional lecture-based teaching. A frequently mentioned educational need was the demonstration of limitations and failure modes of LLMs, including incorrect outputs and hallucinations.

#13: “A few examples of prompting errors […] i.e. preferably show what the negative examples are. I think it’s very, very important to see what the limits are.”

Additional suggestions included live comparisons of different LLMs and incorporation of the patient perspective into educational formats. Participants in earlier training years (≤ 3) more often discussed prompting skills, teaching examples of AI errors and the potential impact of AI on independent thinking, whereas those in advanced training years (≥ 4) expressed more frequently concerns about uncritical reliance on AI and expressed the importance to develop a critical attitude towards AI.

#### Dimensions of AI-related competence

Participants described AI competence as a multidimensional construct (Table 3).

**Table 3:** Perceived competencies and educational needs for the appropriate use of large language models: subcategories, codes and anchor quotes from general practice trainees

**Code**	Description	Anchor Quote
Current state of competence acquisition		
Informal skills acquisition	So far, AI skills have mainly been acquired informally, autodidactically and in collegial exchange (learning by doing). (n = 5: #3, #5, #6, #15, #16)	*#6: "A lot of learning by doing, I would say [...] to deal with AI, to try out different ones, to use different large language models."*
Request for training	Desire for structured AI training in studies and further education. (n = 8: #1, #2, #3, #9, #10, #13, #14, #17)	*#1: "Well, I think many people would have to be taught how to use AI again in a seminar. What are the limitations of AI? How are AI models so roughly structured?"*
Educational preferences and needs		
Practice-oriented, case-based learning	Favoring interactive formats with patient cases, hands-on AI use, and error reflection. (n = 9: #1, #2, #3, #6, #10, #13, #15, #16, #17)	*#6: "i.e. that you really go through different case studies and then show the AI answers."*
Desire for negative examples	Desire to demonstrate negative examples/mistakes and hallucinations in seminars in a targeted manner. (n = 6: #1, #6, #10, #12, #13, #15)	*#13: "A few examples of prompting errors [...] i.e. preferably show what the negative examples are. I think it's very, very important to see what the limits are."*
Dimensions of competence		
Technical expertise	Need to understand basic workings of AI/LLMs, boundaries, privacy, and technical backgrounds. (n = 8: #1, #2, #4, #9, #11, #13, #15, #17)	*#4: "Of course, you have to understand how an AI works. […] So it's quite essential to have certain technical skills."*
Data protection competence	Selection of suitable, medically sound systems; Know what data can be entered; Knowledge of data protection, risks and fields of application. (n = 7: #5, #8, #9, #11, #12, #14, #17)	*#12: "You have to look at what data protection does, what can you enter, what should you not enter."*
Prompting expertise	Input quality as a limiting factor: the ability to use AI in a targeted manner, to formulate appropriate questions and to adapt prompts; Input quality determines result quality. (n = 11: #1, #4, #5, #6, #8, #9, #10, #12, #13, #15, #17)	*#6: "So overall the handling of: How do I write prompts in [...] I want to have an evidence-based result."*
Application expertise	Knowledge of fields of application and AI applications. (n = 11: #1, #5, #6, #8, #9, #11, #12, #13, #14, #15, #17)	*#5: "Probably first of all, it's about an introduction to AIs in general. Which are technically sound, which are not."*
Evaluation competence	Ability to question AI responses, recognize errors, hallucinations, and uncertainties; Ability to assess source quality, assess evidence-based information, and classify AI results. (n = 11: #3, #5, #6, #7, #10, #11, #12, #14, #15, #16, #17)	*#11: "the competencies [...] To critically question information. The information also to understand how the AI works in the end."*
Medical Competence	The prerequisite for the use of AI is clinical expertise and experience. (n = 7: #2, #3, #5, #7, #8, #12, #16)	*#8: "the broader the background knowledge and the clinical experience, the better [...] The broader my background is, the better it is."*

#4: “Of course, you have to understand how an AI works. […] So, it’s quite essential to have certain technical skills.”

Three competency dimensions were mentioned most frequently. Critical evaluation competence comprising the ability to assess LLM-generated information, recognizing errors and uncertainty and evaluating the reliability of underlying sources. Prompting competence referred to the ability to formulate effective queries, with participants pointing out that input quality strongly influenced output quality. Application competence included knowledge of appropriate use cases and awareness of available AI tools.

Beyond these core dimensions, participants named technical competence, including a basic understanding of LLM functioning and limitations, and data protection competence, including the selection of appropriate systems and awareness of permissible data handling. Broad medical expertise and clinical experience were described as essential prerequisites for evaluating AI-generated information appropriately.

The identified competency dimensions and corresponding subcategories are summarized in Table 3.

### Impact of large language models on professional competencies and continuing education

This category captures participants’ perceptions of how AI use reshapes physicians’ skills. The subcategories are organized along the direction of this change: capabilities that AI was perceived to enhance, capabilities at risk of diminishing through reliance on AI and aspects of medical practice regarded as essentially unaffected by AI.

#### Perceived enhancement of clinical work

The most frequently described benefit of LLM-use was support for knowledge acquisition. Participants reported using LLMs to obtain rapid explanations, summarize complex topics and access medical information more efficiently.

#17: “I use it to inform myself, to have things summarized, simply to get information in a time-efficient manner […] that the relief will allow you to take care of the patient a little more again.”

Participants also described relief from documentation-related tasks, including certificates, reports and summaries. In addition, LLMs were perceived to support diagnostic reasoning by broadening diagnostic considerations and improving coverage of rare diseases in general practice. Some participants reported increased efficiency and the possibility of creating more time for patient care. Others described a potential shift in the physician’s role from being primarily a holder of knowledge towards becoming a navigator and evaluator of medical information.

#### Concerns regarding cognitive dependency

The most frequently reported concern related not to practical skills but to cognitive processes. Many participants expressed concerns that excessive reliance on LLMs could reduce independent, critical and analytical thinking, sometimes described as “mental laziness”.

#1: “Well, I hope it doesn’t go in the direction that it reduces our independence to think, which could certainly be possible if you rely completely on AI now.”

Participants also anticipated a potential decline in factual knowledge and memory, partly comparing AI use to reliance on calculators. Some participants described changes in learning behavior, with more targeted, question-driven information acquisition replacing broader forms of learning, such as reading comprehensive texts. In specialties relying heavily on pattern recognition, some participants anticipated a reduced role of human description and increased reliance on AI-supported confirmation and monitoring.

#### Human competencies perceived as difficult to replace

Participants identified several aspects of medical practice that they considered difficult to replace by AI. Interpersonal competencies were stated most frequently, including relationship-building, trust, empathy, contextual understanding, family knowledge and non-verbal communication.

#5: “The whole family history and the psychosocial component, which is so extremely important in general practice. […] I don’t think we could ever replace that with an AI, […] because there the interpersonal relationship is the most important factor.”

Practical skills, including physical examination and sonography, were also considered difficult to substitute.

#### Implications for postgraduate education

Participants expressed mixed views regarding the role of LLMs in postgraduate training. Participants described LLMs as supportive learning resources that enable faster and broader knowledge acquisition and provide an accessible source of information. In contrast, some participants perceived limited influence on structured postgraduate education, pointing out the continuous importance of experienced human supervisors. They also referred to selective learning behavior in the context of postgraduate training and one participant reflected on the potential development of dependency through intensive AI use.

The identified subcategories are summarized in Table 4.

**Table 4:** Perceived impact of large language models on clinical competencies and postgraduate learning: subcategories, codes and anchor quotes from general practice trainees

Code	Description	Anchor Quote
Enhanced capabilities		
Support in the acquisition of knowledge	AI supports doctors in acquiring knowledge, explaining complex issues and quickly acquiring new content. (n = 10: #1, #5, #6, #8, #9, #11, #12, #13, #14, #16)	*#5: "Acquisition of expertise maybe, yes. […] because one would not have researched in the depths of the specialist literature at all."*
Creation of texts and documentation	Text/writing tasks (certificates, reports, e-mails, summaries, certificates). (n = 7: #1, #3, #5, #6, #13, #14, #15)	*#13: "It's a great support, especially with bureaucratic documentation activities. That's what I mainly use it for."*
Support in diagnostics	AI expands diagnostic possibilities, supports differential diagnoses and helps with complex cases; increasing diagnostic breadth, better coverage of rare diseases ("zebras"). (n = 5: #1, #2, #13, #15, #17)	*#1: "Yes, so it could increase the diagnostic breadth a bit [...], in order to perhaps also cover a bit rarer things in the family doctor's office."*
Increased efficiency	AI enables faster work processes, reduces administrative burdens and thus creates quantitative empowerment and more time for patients. (n = 3: #1, #6, #17)	*#17: "I use it to inform myself, to have things summarized, simply to get information in a time-efficient manner […] that the relief will allow you to take care of the patient a little more again."*
Increase in the function of primary care physicians as a filter and guide	AI is shifting the focus of medical activity from knowledge transfer to classification, filtering and control of care. (n = 3: #8, #11, #13)	*#13: "But I believe that in the long term the doctor will be more of a knowledge pilot than the knowledge holder."*
AI more empathetic than physicians	AI systems are sometimes perceived as more empathetic compared to doctors. (n = 1: #14)	*#14: „[…] in some cases, AIs are actually more empathetic than some doctors. So many doctors already have burnout, can no longer really listen to the patients. So I think AI will take over a lot in the future."*
Diminishing capabilities		
Loss of medical factual knowledge	Danger of a decrease in medical factual knowledge and retention. (n = 8: #2, #5, #6, #7, #8, #10, #11, #14)	*#14: "My memory decreases, [...] because I know that I can look it up at any time."*
Loss of critical thinking	Danger of losing the ability to think independently/critically/analytically ("laziness to think"). (n = 11: #1, #2, #3, #5, #6, #9, #10, #12, #14, #15, #17)	*#1: "Well, I hope it doesn't go in the direction that it reduces our independence to think, which could certainly be possible if you rely completely on AI now."*
Loss of research skills and selective learning behaviour	There is a risk that research skills will decrease and knowledge will be acquired in a targeted and question-related manner, while broad, casual learning (e.g. when reading chapters across) will decrease. (n = 4: #7, #8, #10, #16)	*#7: "I think you learn differently. So you just learn so selectively [...] And I think it's more specific what you learn there and maybe not so broad."*
Loss of human pattern recognition	Pattern recognition subjects (radiology, endoscopy, etc.) are losing their diagnostic role to AI. (n = 3: #2, #4, #14)	*#2: "Yes, I believe that professions that are based on pattern recognition, for example pathology, histology, radiology, [...] in the future, significantly more will have a confirming and monitoring function and less will diagnose themselves."*
Aspects unaffected by AI		
Interpersonal aspects irreplaceable	Interpersonal relationships, trust, empathy, experienced anamnesis, context and non-verbal communication are irreplaceable. (n = 11: #1, #2, #3, #5, #6, #7, #8, #10, #12, #13, #16)	*#3: "Your family doctor just sees the complete anamnesis. He knows the patient, the family behind it. He knows whether the patient is anxious. […] The AI can't do that, it throws out information – unfiltered."*
Practical skills irreplaceable	Practical skills such as physical examination and sonography are not replaceable. (n = 5: #3, #5, #6, #12, #13)	*#3: "Home visits, physical examinations [...] whether it's an ultrasound or listening to the lungs, I think that shouldn't be lost and AI can't replace that."*
Little influence on the structure of postgraduate education	Little to no influence on structured training. (n = 5: #2, #3, #10, #11, #15)	*#2: "With regard to structured postgraduate education, no (influence) at all."*

### Influence of large language models on diagnostic uncertainty

This category examines how AI affects the experience and management of uncertainty in clinical practice, a central characteristic of general practice. The subcategories reflect the direction of this effect, comprising mechanisms through which AI was perceived to decrease uncertainty, those through which it increases uncertainty and factors that modulate the extent and direction of this influence.

#### Subjective reduction of uncertainty

Most participants described LLMs as reducing subjective diagnostic uncertainty, primarily by confirming an already established clinical assessment. Some participants described LLMs as a means of managing uncertainty, particularly when queries were specific and clearly formulated. However, participants also named the risk that increased confidence may not necessarily reflect increased accuracy.

#6: “There may sometimes be a false sense of security when [AI] comes up with the same [idea] as I do now […] it reduces some of the uncertainty, but with such an aftertaste.”

Participants described that LLMs could produce responses with high apparent certainty and only reveal limitations when actively challenged, potentially leading to reassurance exceeding actual reliability.

#### Additional sources of uncertainty

Participants also identified situations in which LLM use could increase uncertainty. Patients’ independent use of LLMs was described as an additional source of uncertainty requiring clarification and discussion during consultations.

#15: “On the patient side, I would say that there is a lot of uncertainty that the doctors then have to endure.”

Furthermore, participants described a potential broadening of diagnostic possibilities through LLMs, which could contribute to excessive investigations or a “shotgun” approach to diagnostics.

#### Factors influencing the effect of LLMs on uncertainty

Participants stated that the effect of LLM use on uncertainty depended on how the tools were used. Non-specific prompts were perceived as more likely to increase uncertainty, whereas targeted questioning was associated with more useful responses.

#17: “You do have to phrase the prompt correctly. If you just put in a plain question, it actually also increases the uncertainty And if you know how to ask a question, you also get better answers.”

In situations with high uncertainty, participants continued to prefer discussion with experienced colleagues and trainers, whose clinical judgment was generally trusted more than LLM outputs. They also argued that uncertainty is an inherent characteristic of GP that cannot be eliminated by technology. One participant additionally highlighted confirmation bias as a potential risk, as suggestive prompts may reinforce existing hypotheses by generating responses perceived as confirming initial assumptions.

The identified subcategories are summarized in Table 5.

**Table 5:** Perceived influence of large language models on diagnostic uncertainty: subcategories, codes and anchor quotes from general practice trainees

Code	Description	Anchor Quote
Decreased uncertainty		
AI reduces uncertainty	AI is perceived as an opportunity to reduce uncertainties and gain more certainty in medical decisions. (*n* = 11: #1, #3, #5, #7, #8, #9, #10, #11, #12, #15, #16)	*#7: "I think it rather lowers uncertainty. […] if the AI sees it the same way, I'd say, you'd feel more vindicated."*
AI to cope with uncertainty	AI is actively used as a strategy to deal with existing diagnostic uncertainty in a targeted manner, for example through precise, specific inquiries instead of blindly trusting the answer. (*n* = 1: #10)	*#10: "Ideally, it should reduce uncertainty here. […] If you don't blindly trust what it spits out, but state very specifically exactly what you want to know."*
False security	AI can reduce perceived uncertainty, although the answer may be incomplete or incorrect; AI responds with (pseudo) certainty and only admits errors after contradiction. (*n* = 9: #2, #3, #5, #6, #10, #11, #13, #14, #15)	*#6: "There may sometimes be a false sense of security when [AI] comes up with the same [idea] as I do now [...] it reduces some of the uncertainty, but with such an aftertaste."*
Increased uncertainty		
AI increases patient uncertainty	Patient-side AI use creates additional uncertainty that doctors have to absorb. (*n* = 4: #7, #8, #15, #17)	*#15: "On the patient side, I would say that there is a lot of uncertainty that the doctors then have to endure."*
AI increases uncertainty due to overdiagnosis	AI can also increase uncertainty: overdiagnosis/oversupply. (*n* = 4: #8, #13, #15, #17)	*#17: "So in some cases, the AI spits out significantly more diagnostics than would probably be necessary now."*
Modulation of uncertainty		
Uncertainty depends on prompting skills	The effect depends on the use (non-specific questions increase uncertainty). (*n* = 2: #10, #17)	*17:“You do have to phrase the prompt correctly. If you just put in a plain question, it actually also increases the uncertainty. And if you know how to ask a question, you also get better answers.”*
Discussion with colleagues in case of uncertainty	Discussion of uncertain cases continues to be discussed with trainers/colleagues. (*n* = 6: #3, #5, #7, #8, #10, #16)	*#5: "I would like to say that I would get a specialist Dixit on this. […] somehow I trust more in the experience of my colleagues."*
Uncertainty is part of general medicine	Uncertainty is part of general medicine and must continue to be endured/carried. (*n* = 2: #6, #11)	*#11: "You can't clarify every diagnosis down to the end and down to the smallest detail, often, but there is also diagnostic uncertainty. And of course, as a doctor, you have to wear them somewhere."*
Confirmation Bias	Confirmation bias: AI provides the most appropriate, not the right answer. (*n* = 1: #2)	*#2: "in the sense of confirmation bias, if you have an idea and then you ask the AI and you enter any questions suggestively, the AI always tries to provide the most suitable answer and not necessarily the right one, then you will often see yourself confirmed, even if your own idea is not right or not the only right one."*

### Ethical and moral aspects of large language models

This table summarizes the ethical and moral considerations participants associated with AI use in general practice. The subcategories span three interrelated areas: the conditions under which AI use was considered ethically acceptable, the ethical concerns raised regarding its use and the question of how responsibility for decisions is allocated between physicians and AI systems.

#### Ethical framing of LLM use

Most participants considered the supportive use of LLMs ethically acceptable when applied critically and responsibly. Participants commonly described LLMs as ethically neutral tools, with ethical implications depending on the way they were used by physicians.

#16: “Well, I always thought it was like a tool. […] So I don’t think AI has any morals or ethics, honestly. I think that’s what stays completely with us.”

Direct physician–patient interaction and shared decision-making in GP were perceived as factors that currently limit ethical concerns associated with LLM use.

#### Ethical risks and governance concerns

Data protection was the most frequently mentioned ethical concern. Most participants named anonymous information or complete avoidance of patient-identifiable information as a prerequisite for responsible use and as part of their professional duty of care.

#5: “Of course, you have to be careful that you do all this anonymously and don’t let any patient data flow into it in any way So first of all, with regard to the data protection of the patient.”

Participants also expressed concerns regarding transparency, independence and traceability of LLM outputs. Unclear data sources, limited insight into system control and potential commercial interests were described as factors, which make ethical deliberation particularly challenging. Additional concerns included ecological impact related to resource consumption, potential disadvantages for patient groups, copyright issues related to training data and dependency on technical infrastructure. Several participants emphasized boundaries regarding delegation of tasks. Value-based or existential decisions concerning patients were considered inappropriate for delegation to AI systems.

#### Responsibility and accountability

Participants showed strong consensus that medical responsibility could not be transferred to LLMs. Referring to an AI-generated recommendation (“The LLM said so”) was not considered as justification for clinical decisions. However, participants simultaneously described a perceived relief associated with LLM support. Some participants expressed concerns that AI assistance could contribute to reduced feelings of responsibility or externalization of decision-making.

Participants described LLMs as providing an “outside perspective”, which could make decisions feel easier while leaving professional responsibility unchanged. Several participants described this discrepancy between perceived and actual responsibility as potentially misleading. One participant suggested that manufacturer liability would be a prerequisite for genuine shared responsibility.

#6: “If at some point something is approved and a manufacturer really says: […] okay, I give a probability of 99% that this is correct and accept the legal responsibility for it, then maybe, but otherwise it is definitely not possible at the moment.”

The identified subcategories are summarized in Table 6.

**Table 6:** Perceived ethical and moral implications of large language models in general practice: subcategories, codes and anchor quotes from general practice trainees

Code	Description	Anchor Quote
Ethical acceptability		
AI as a tool ethically justifiable	Ethically justifiable as an aid, if used critically; low ethical concerns in a 1:1 GP setting (participatory decision-making); AI is regarded as a neutral instrument, and the ethical assessment is only created by the person who uses it. (*n* = 8: #1, #2, #3, #5, #10, #14, #16, #17)	*#16: "Well, I always thought it was like a tool. [...] So I don't think AI has any morals or ethics, honestly. I think that's what stays completely with us."*
Ethical concerns		
Data protection and anonymization as a prerequisite	The use of AI is only considered ethically justifiable on the condition that no patient-related data is entered; Anonymization is described as one's own duty of care. (*n* = 10: #1, #2, #3, #4, #5, #6, #10, #12, #13, #14)	*#5: "Of course, you have to be careful that you do all this anonymously and don't let any patient data flow into it in any way. So first of all, with regard to the data protection of the patient."*
Doubts about the independence and transparency of AI	Lack of transparency: unknown sources, interests and control behind the AI; Concern about misinformation and commercial influence (pharmaceuticals, advertising) on AI responses. (*n* = 9: #1, #2, #6, #7, #8, #10, #13, #15, #17)	*#13: "In the sense of, simply, how independent is an AI really? How is it controlled? […] So you never really know what the background is. What's behind it. What the actual interests are."*
Environmental concerns	Ecological concerns (electricity, water, energy consumption). (*n* = 3: #1, #13, #15)	*#13: "And what is also a point for me is the ecological aspect with the high water consumption. That's why I don't use it so much privately."*
Discrimination against patient groups	Risk of disadvantage of patient groups. (*n* = 1: #2)	*#2: "If you don't calculate this for the individual patient, but for entire population groups, it may be that AI says with a utilitarian ethics in care, [...] certain people and groups should not receive care or only to a limited extent in order to save resources. I actually have concerns about that."*
Copyright of the training content	Copyright/remuneration of the creators of the training content. (*n* = 1: #15)	*#15: "There are a lot of problems with copyright, of course. How are the people who created the material on which the AI is built compensated?"*
Risk of dependency in the event of system failure	Dependency/failure risk for the entire system (server failure). (*n* = 1: #6)	*#6: "And then, of course, a big risk, I'll say, to what extent you want to make yourself dependent on it as a society, as a health system and so on. […] if something should fail now, be it any server, Internet something and everything runs on it, I also have a huge problem."*
Responsibility		
Responsibility remains with the physician	Responsibility remains with the doctor and cannot be delegated to the AI. (*n* = 11: #3, #5, #6, #9, #11, #12, #13, #14, #15, #16, #17)	*#16: "In the end, we are the decision-makers too. And that's also our job, I think."*
Sense of shared responsibility	Concern that AI support will make doctors feel less responsible or externalize decisions. (*n* = 7: #5, #6, #7, #8, #9, #11, #15)	*#7: "It could be that you feel less responsible as a doctor [...] it could also be that the AI is then put in charge."*
Shared responsibility only if AI companies assume liability	Assumption of liability by manufacturers as a prerequisite for genuine sharing of responsibility. (*n* = 1: #6)	*#6: "If at some point something is approved and a manufacturer really says: [...] okay, I give a probability of 99% that this is correct and accept the legal responsibility for it, then maybe, but otherwise it is definitely not possible at the moment."*

## Discussion

To our knowledge, this is the first qualitative study exploring AI use and its perceived impact on clinical reasoning among postgraduate GP trainees. Our findings indicate that AI is commonly used in clinical practice while structured AI education is currently lacking within postgraduate training. Trainees show great interest to develop competencies for the responsible use of AI in clinical practice. These findings suggest that AI-related competencies should become an integral part of curricula in postgraduate medical education.

### AI as augmentation rather than replacement of clinical expertise

AI is increasingly used in GP [23, 24]. Most participants described AI as a supportive tool that can complement physicians’ clinical expertise. They did not perceive AI as an autonomous decision-maker but rather as a resource that may enhance diagnostic reasoning and facilitate access to medical knowledge. As primary care is characterized by high levels of complexity, clinical uncertainty, multimorbidity and the influence of social determinants of health [3, 25], not all clinical decisions can be effectively automated by AI, underscoring the continued importance of physicians’ clinical judgment and contextual decision-making [8].

These perspectives are consistent with current discussions on the role of AI in medicine [26], suggesting that the greatest benefit of AI may arise from combining computational capabilities with human expertise rather than attempting to replace physicians. Topol et al. [27] point out, that future medical practice will depend on the integration of AI-supported analysis with human abilities, such as empathy, contextual understanding and clinical judgement. In our study, participants similarly described AI as valuable for expanding perspectives and supporting decision-making, while maintaining the physician’s role in interpreting information and applying it to individual patient situations.

The increasing availability of AI-generated information may shift the competencies required from physicians. While access to information becomes increasingly effortless, the ability to evaluate relevance, recognise limitations and integrate information within the clinical context becomes more important. This shift from physicians primarily acting as knowledge providers towards knowledge interpreters [28] represents an important consideration for future medical education as well as the physician’s role in general.

### Impact of AI on clinical reasoning

The findings about clinical reasoning suggest that participants may integrate AI into existing reasoning processes rather than fundamentally change their approach to clinical decision-making. A common approach was to first develop their own diagnostic hypotheses and then use AI to challenge or complement their thinking in this context. This goes along with the concept of augmented intelligence, where AI enhances rather than substitutes human expertise [27]. Some participants reported that AI primarily accelerates existing decision-making processes and does not change it relevantly.

Participants also identified potential risks. Due to the broad scope of GP and limited opportunities for immediate peer consultation, AI can be an attractive source of support but could also increase the risk of uncritical acceptance of AI-generated information. They further raised concerns that extensive use of AI could lead to cognitive offloading [29] and reduce opportunities to fully develop independent clinical reasoning skills. While automation bias [30] or sycophancy was not described as a current major problem, some participants acknowledged that plausible and convincing AI-generated responses could influence judgement.

This creates a relevant challenge for postgraduate education: while AI may support trainees by reducing cognitive workload, over-reliance on AI-generated information could limit opportunities to develop independent clinical reasoning skills.

#### AI as supportive technology: maintaining professional responsibility

Participants consistently described AI as a tool that can ethically support physicians’ clinical decision-making. Responsibility for diagnostic and therapeutic decisions was regarded as inseparable from the physician’s professional role, no matter whether AI was involved.

They perceive AI as morally neutral and state that responsibility, accountability and ethical judgment remain human tasks, while AI may alter the subjective experience of responsibility.

This view aligns with international statements saying that the use of AI in healthcare must preserve human oversight and accountability [27, 31]. Ethical concerns raised by participants focused less on abstract questions of AI ethics than on practical issues encountered in everyday clinical work. Data protection was the most frequently discussed concern, accompanied by uncertainty and intransparency about the origin of AI-generated information. These findings reflect ongoing discussions about the prerequisites for trustworthy AI in healthcare, particularly the need for transparent governance and clearly defined accountability [32, 33].

Interestingly, while participants firmly rejected the idea of delegating clinical responsibility to AI, some acknowledged that AI-supported recommendations could subtly influence their own sense of responsibility. The potential for unintended AI influence and overreliance on AI underscores the challenges associated with changes in clinical reasoning and reinforces the importance of developing a professional attitude and professional identity formation within postgraduate training. Postgraduate education needs to encourage critical reflection on professional responsibility, appropriate reliance on AI and ethical decision-making in AI-supported clinical practice [34].

#### Implications for postgraduate medical education

Participants consistently described AI as a tool that was already part of their daily clinical practice and none of them had received structured postgraduate training on the use of AI [16] in clinical practice and reported having learned to use AI through informal trial and error. Furthermore, participants did not report on structured exchange about use of AI in daily practice and expressed a need for exchange about how to use AI. Participants also identified a possible risk: The easy access to AI-based support may compensate for gaps in clinical supervision and could unintentionally reinforce existing problems in training environments and postgraduate medical training in Germany. They expressed a need for structured AI education, emphasising a relevant gap in the current competency-based postgraduate training curriculum for GP in Germany [35].

Clinical reasoning and critical appraisal of AI-generated information will become increasingly important as physicians are required to evaluate AI recommendations, especially in GP [36]. These findings indicate that postgraduate training should prepare physicians to use AI effectively to improve patient care while understanding its capabilities and limitations and applying it responsibly. The results of our qualitative study broadly align with recently proposed AI competency frameworks [37] and three core domains of competence can be identified: (1) the practical clinical application of AI tools, (2) the critical appraisal of AI-generated information and (3) ethical and professional responsibility in AI-assisted clinical practice. Participants emphasised the importance of understanding the limitations of AI. Educational programmes could address this by using clinical examples of AI errors and inappropriate reliance on algorithmic recommendations. Such approaches may foster reflective practitioners [38] who integrate AI thoughtfully into clinical decision-making [7].

This study suggests that postgraduate medical education should focus on how to critically evaluate and responsibly integrate AI-supported clinical reasoning longitudinally across undergraduate, postgraduate and continuing medical education using an approach that combines repeated practice with structured opportunities for reflection.

### Strengths and limitations

A major strength of this study is the inclusion of GP trainees at different stages of training with relevant clinical and educational experience. Their perspectives provide valuable insights into current experiences with AI and educational needs during postgraduate training. Several limitations should be considered. While the findings may be relevant for most postgraduate GP trainees, transferability to other contexts and healthcare systems may be limited. AI technologies are rapidly developing. Therefore, the relevance of the findings may change as AI systems may change within the next years. Social desirability bias may have influenced participants’ responses, particularly regarding their disclosure of AI use. Unfortunately, no participants without prior experience using AI tools and only two with little AI use could be recruited, which might limit our results.

## Conclusion

AI is already being used by GP trainees as a supportive tool for clinical reasoning, knowledge acquisition and clinical decision-making. Responsible AI integration requires competencies in critical appraisal of AI outputs, clinical application, prompting, data protection and ethical decision-making, alongside strong medical knowledge and clinical reasoning skills. Postgraduate medical education should therefore incorporate AI-related competencies to enable future general practitioners to critically assess, appropriately use and responsibly integrate AI while maintaining professional judgment and accountability.

## Supplementary Information

Supplementary Material 1.

Supplementary Material 2.

## Data Availability

The datasets presented in this article are not readily available because as required by the European Data Protection law the participants were informed of the privacy policy and agreed that the pseudonymized data would be available to the project team and would be stored at the study center. Request to access the datasets should be directed to Simon Schwill (simon.schwill@med.uni-heidelberg.de).

## Abbrevations

GP: General Practice
GPS: General Practitioners
KW: Kompetenzzentrum Weiterbildung (German) = Competence Center for postgraduate medical education
