# Supplementary material for: Artificial intelligence and clinical reasoning - a qualitative study with general practice trainees in Germany
Source: BMC Med Educ. 2026 Nov 14;26:1862. doi: 10.1186/s12909-026-10736-3 (PMC13769186; doi:10.1186/s12909-026-10736-3)
Supplement: Supplementary file 2 [file 12909_2026_10736_MOESM2_ESM.docx]

**Artificial Intelligence and Clinical Reasoning in General Practice**

Pseudonym: Date:

**INTERVIEW GUIDE – INDIVIDUAL INTERVIEWS**

Research questions

**Decision-making**

To what extent does the use of artificial intelligence change decision-making and communication in the general practice consultation? Which competencies are required when working with artificial intelligence? What implications arise for postgraduate training in general practice?

1) **Study information and informed consent**
2) **Questionnaire**

| Age |  |
| --- | --- |
| Gender | □ Male □ Female □ Diverse |
| Training status | □ Physician in postgraduate training □ Specialist in lateral entry |
| Year of postgraduate training |  |
| Year of specialist examination |  |
| How often do you use AI professionally? | □ daily  □ several times per week  □ once per week  □ less than once per week  □ never, but generally interested  □ never, no interest |
| How would you rate the degree of digitalization at your workplace? | □ little digitalized: mostly paper-based  □ partially digitalized  □ largely digitalized  □ fully digitalized |

3) **Interview:**

| **Topic** | **Question** |
| --- | --- |
| Thank you very much for your willingness to take part in this interview.  For the recording, I would like to make sure that you have received the information sheet, that all your questions about the study have been answered, and that you consent to the video and audio recording of the interview.  Please answer yes or no. | |
| Use of AI | What role does AI play in your daily life?  Follow-up questions:   - To what extent does AI support you in your medical work? - Which applications do you or your practice use specifically? - Do you have a paid account, and if so, which one? |
| clinical reasoning | To what extent are physicians, in your opinion, influenced by AI in clinical reasoning?  Follow-up questions:   - Are there any particularities among general practitioners in this regard? - To what extent does AI influence your personal decision-making in the consultation? |
| Use and evaluation | How do you use and evaluate statements made by AI?  Follow-up questions:   - To what extent have you had experiences with incorrect AI statements (“hallucinations”)? - How do you deal with contradictions between your assessment and the assessment of AI? |
| Uncertainty | To what extent does AI influence diagnostic uncertainty in general practice work? |
| Competencies | Which competencies do general practitioners need for the use of AI?  Follow-up questions:   - How can these competencies be learned? - How would you design a course for physicians in postgraduate training in general practice that teaches how to work with AI? |
| Postgraduate training | To what extent does AI have an influence on your postgraduate training? |
| Medical skills | To what extent will medical skills change in the long term through the use of artificial intelligence?  Follow-up questions:   - Which skills will decline? Which will increase? What could AI enable? |
| Ethics and morality | How do you evaluate AI from an ethical and moral perspective?  Follow-up questions:   - To what extent could AI influence the perceived responsibility in clinical decisions? |
| Additional remarks | Would you like to add anything? |
|  | Then we will end the interview recording. Thank you very much! |
